# Familial amyloidotic polyneuropathy with muscle, vitreous, leptomeningeal, and cardiac involvement: Phenotypic, pathological, and MRI description

**DOI:** 10.4103/0972-2327.64642

**Published:** 2010

**Authors:** D. K. Prashantha, Arun B. Taly, Sanjib Sinha, T. Chikkabasavaiah Yasha, Narayanappa Gayathri, J. M. E. Kovur, Joy Vijayan

**Affiliations:** Departments of Neurology, Neuroimaging and Interventional Radiology, Bangalore, India; 1 Department of Neuropathology, NIMHANS, Bangalore, India

**Keywords:** Amyloidosis, cardiomyopathy, familial amyloidotic polyneuropathy, leptomeningitis, myopathy, neuropathy, vitreous deposits

## Abstract

Familial amyloidotic polyneuropathy (FAN type 1) is a rare systemic disease that causes severe and disabling peripheral neuropathy. We describe the phenotypic, radiological, and pathological characteristics of a patient with familial amyloid polyneuropathy type 1 who had evidence of motor-sensory-autonomic neuropathy, ocular vitreous deposits, diffuse leptomeningeal involvement, and hypertrophic cardiomyopathy. Muscle involvement, an infrequently reported feature, was also observed. Early recognition of the disease has significant therapeutic implications.

## Introduction

Familial amyloidotic polyneuropathy (FAN) is a form of fatal hereditary amyloidosis due to mutations in various genes. Based on the proteins accumulated, there are four types. Type 1, the most common form, is due to a mutation in transthyretin (TTR) and is characterized by peripheral and autonomic neuropathy, as well as vitreous and leptomeningeal involvement.[1] Primary amyloidosis is more prevalent than FAN but it mainly involves the small fibers of peripheral nerves or causes the carpal tunnel syndrome.[2]

We report a patient of FAN type 1 with peripheral nerve, autonomic nervous system, muscle, vitreous, leptomeningial, and heart involvement and discuss the diagnostic dilemmas.

## Case Report

A 23-year-old male graduate presented with insidious onset, gradually progressive weakness and numbness of both lower limbs and recurrent vomiting of 16 months' duration. He had had blurring of vision, urgency and hesitancy of micturition, erectile dysfunction, and weight loss over the last 6 months. At the beginning of his illness he had been treated at a peripheral center, where he was investigated for recurrent vomiting with ultrasound of the abdomen and upper gastrointestinal endoscopy. These tests did not reveal any abnormality and his vomiting subsided with antiemetics and proton pump inhibitors. There was no other significant history in the past. He had been born to nonconsanguineous parents and there was no family history of systemic or neurological illness.

The patient weighed 35 kg and had pallor, bilateral pedal edema, and thyromegaly. His vital signs were stable. Ocular examination revealed dilated (8 mm) and nonreactive pupils and vitreous deposits; his visual acuity was 6/24 in both the eyes. The fundi could not be visualized clearly. He had hypotonia in all four limbs, moderate weakness of handgrip and of the intrinsic muscles of the hand on both sides, and profound weakness of the dorsiflexors and plantar flexors of the ankle and toes (MRC: 1/5). Muscle stretch reflexes were normal in the upper extremities. Knee jerks were brisk but ankle jerks were absent. There was no plantar response. He had graded sensory impairment in his lower limbs up to the knee for all sensory modalities. The skin below the knees was dry and shiny, with hair loss. The rest of the examination was unremarkable.

The complete hemogram revealed anemia (Hb: 9 gm/dl) and a rapid ESR (74 mm in the 1^st^ hour). Routine analysis of urine was normal. Serum biochemistry showed a low serum albumin (3.3 gm/dl; normal > 3.5 gm/dl), while glucose, renal and liver function tests, and electrolytes were normal. ELISA test for HIV and investigations for vasculitis, including serum ANA, ANCA, dsDNA, LE cell, and RA factor, were negative. The cerebrovascular fluid (CSF) was acellular, with elevated protein of 170 mg/dl (normal: <45.0 mg/dl) and normal glucose (66 mg/dl). The urine was negative for Bence-Jones proteins. Serum electrophoresis and immunofixation, and the bone marrow were normal. Nerve conduction study of the median, ulnar, common peroneal, and sural nerves showed unexcitable motor and sensory nerves in all the four extremities. Autonomic function tests showed absent sympathetic skin response (SSR) in all four limbs and postural drop of blood pressure (54 mmHg systolic and 9 mmHg diastolic). The heart rate response to deep breathing and standing was also impaired. Routine ECG was within normal limits and 2D-echocardigram revealed a nonobstructive cardiomyopathy. The possibilities of mitochondrial neurogastrointestinal encephalomyopathy (MNGIE), familial amylodotic neuropathy (FAN), and chronic inflammatory demyelinating neuropathy (CIDP) with autonomic involvement was considered.

**Figure 2:**
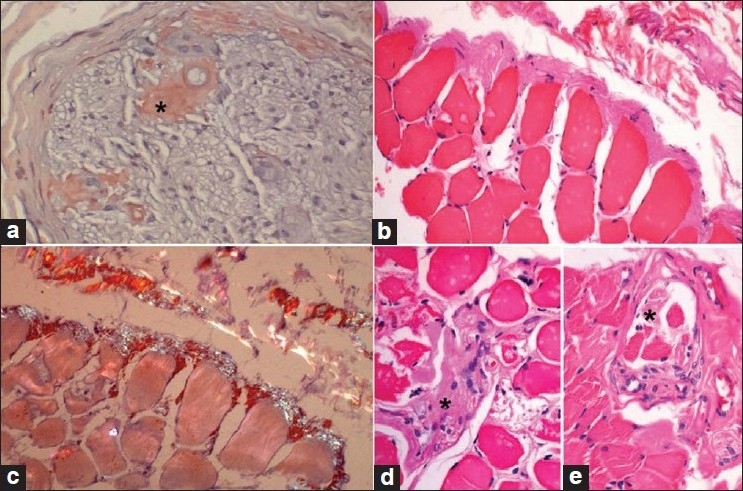
Nerve and muscle biopsy. (a) Sural nerve biopsy showing part of one fascicle with large (*) and small amorphous endoneurial perivascular deposits of amyloid. (b–e) Muscle biopsy; abundant perimyseal and subperimyseal amyloid at the periphery of a fascicle, partly extending between the myofi bers (b); deposits are congophilic and display apple-green birefringence under the polarizer (c); amyloid deposits are also seen in the intramuscular nerve twig (*) (d) and in the muscle spindle (*) (E). (a and c): Congo red stain; c viewed under polarizer; b, d, and e: H and E stain; a–c: ×160; d and e: ×320, original magnifications)

Sural nerve and biceps muscle biopsies showed multiple amyloid deposits. In the nerve it was abundant in the subperineurial and perivascular areas of the endoneurium [Figure 2a] and, to a lesser extent, in the epineurial vessel walls. Myelinated fiber loss was almost total. In the muscle the amyloid deposits were typically maximal at the periphery of the fascicles surrounding individual myofibers and in the perimysium [Figure 2b and c]. In addition, accumulations were seen within intramuscular nerve twigs [Figure 2d], in consonance with their known predilection for this site, and also in muscle spindles [Figure 2e], probably as an extension of the intraneural deposition. The deposits revealed characteristic apple-green birefringence on Congo red staining and polarization microscopy. Immunostaining for kappa and lambda light chains was negative.

**Figure 1:**
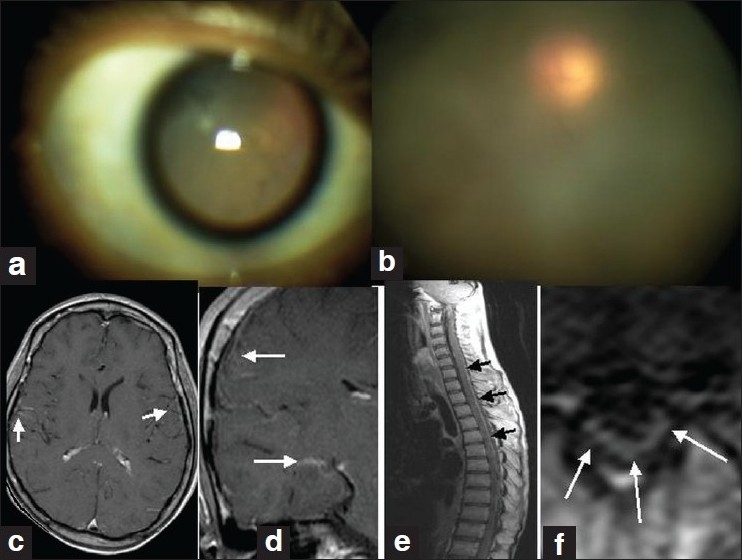
a–e: (a) Dilated pupil; (b) hazy vitreous; (c and d) postcontrast axial and coronal T1W MRI of brain showing leptomeningeal enhancements (arrows); (e and f) post-contrast sagittal and axial T1W MRI of spine with extensive meningeal enhancement

**Figure 3:**
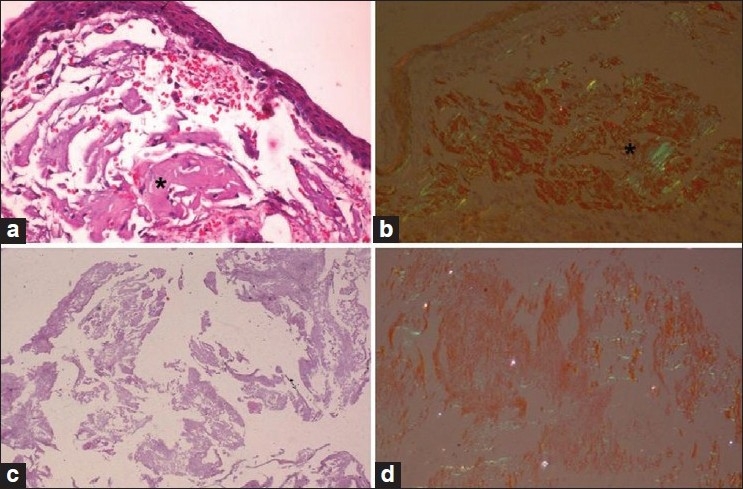
Conjunctival biopsy and vitreous material. (a) Conjunctival biopsy with large amyloid aggregates in the subepithelial tissue (*); (b) exhibiting typical congophilia and birefringence under the polarizer (*); (c) fluffy vitreous material; (d) displaying congophilia. (a and c: H and E stain; b and d: Congo red; a–d ×80 original magnification)

MRI of the brain and spinal cord revealed extensive leptomeningeal enhancement in the post-gadolinium T1-weighted sequences. There was no signal alteration in the brain or spinal cord parenchyma [Figure 1c‐f]. The patient underwent vitrectomy, following which there was improvement in his vision. Conjunctival biopsy and vitreous material also showed evidence of amyloidosis [Figure 3a‐d]. After 6 months, there was slight improvement in vision without any changes in the other parameters.

## Discussion

Primary amyloidosis is due to deposition of immunoglobulin light chain, is associated with paraprotein, and often manifests with the carpal tunnel syndrome rather than symmetrical and extensive sensory-motor-autonomic neuropathy. Our patient had evidence of motor-sensory-autonomic neuropathy, ocular vitreous deposits, diffuse leptomeningeal involvement, asymptomatic myopathy and hypertrophic cardiomyopathy, and normal serum electrophoresis and immunofixation. A diagnosis of FAN could be confirmed by detection of mutation and /or immunohistochemistry of the tissue with anti-human TTR antibody.[1] These investigations could not be carried out in our case due to lack of facilities. TTR is a normal tetrameric protein that is involved in the transport of thyroxine and retinol-binding protein. It is located in chromosome 18 and is produced mainly in the liver, retinal pigment epithelium, choroid plexus, and yolk sac. It has an autosomal dominant pattern of inheritance, and a family history may not be present in more than 50% of cases.[1] More than 100 causative mutations have been described, with the commonest being FAP ATTR V30M.[3] Familial amyloidosis was first described in 1929 by DeBruyn and Stern and constitutes about 2–4% of all patients of amyloidosis.[1] There are four major types of FAN, depending on the type of aberrant proteins accumulated (e.g., TTR, ApoA1, or gelsolin). Type 1 FAN is the commonest and presents with peripheral and autonomic neuropathy, and vitreous and leptomeningeal involvement. Type 2 is the restricted form of type 1 and manifests with carpal tunnel syndrome, but no autonomic involvement. Type 3 is characterized by duodenal ulcers and early renal involvement. Type 4 manifests with cranial nerve involvement and corneal dystrophy.[4] Our patient had features of type 1 FAN.

TTR-related FAN was first described from Japan and certain European countries. It is an invariably progressive disease, with a mean age of onset of 35.3 years; it reaches the terminal stage in around 10 years.[3] The dominant phenotype shows sensory disturbances (100%), motor weakness and wasting (80%), autonomic dysfunction (>80%), and leptomeningeal and ocular involvement (20%).[2] Cerebral involvement is rare but, when present, manifests with cerebral infarction and hemorrhage, dementia, hydrocephalus, seizures, and pyramidal signs.[5] This might be due to amyloid deposits in the leptomeningeal and cerebral vessels causing ischemia and impaired autoregulation.[4] These patients have evidence of multisystem involvement, including cardiac and renal involvement, which often leads to early death. Survival is for 10–15 years. MRI studies in these patients have shown enhancement of the leptomeninges, labyrinth, dentate ligament, and vitreous body following gadolinium administration.[6,7] The present case did have extensive enhancement of leptomeninges and vitreous. Liver transplantation has been tried to halt the progression of the disease.

Our patient had demonstrable amyloid deposition in nerve, muscle, conjunctiva, and vitreous. Spuler *et al* (1998) studied 13 adults with muscle weakness of 3 months' to 4 years' duration with a diagnosis of systemic amyloidosis and demonstrated congophilic deposits around blood vessels and muscle fibers in all the muscle specimens. The authors stressed the importance of fluorescent Congo red stain as a routine procedure in the diagnosis of amyloidosis.[8] Muscle involvement was also studied in another series of 35 patients with amyloid neuropathy, and 9 of the 25 patients of FAN had muscle involvement.[9] Amyloid myopathy in a 62-year-old man with progressive proximal weakness has been reported. This diagnosis should always be a consideration in adults with progressive neuromuscular weakness of uncertain cause.[10] Neuropathy is attributed to preferential deposition of amyloid in the sensory and autonomic ganglia, which causes axonal degeneration. Also, there is endoneurial edema due to the amyloid, resulting in ischemia. Nerve biopsy in the early stages may reveal a relatively greater reduction of small myelinated and unmyelinated fibers. In the advanced stages of the illness, large myelinated fibers also decrease. The largest and most numerous amyloid deposits are seen within the nerve fascicles.

This is one of only two case reports of FAN from India with ocular, skeletal muscle, cardiac, and leptomeningeal involvement; the other case, reported from north India, was that of a lady presenting with sensory/motor neuropathy, without any systemic involvement.[11] A high index of suspicion, with careful attention to clinical clues and histological characteristics are essential for accurate diagnosis. Genetic analysis is essential for confirmation. Symptomatic treatment for dysautonomia and visual disturbances, along with rehabilitative measures, can improve the quality of life in these patients.
